# Efficacy of Aqueous Moringa Oleifera Leaf Extract as a Natural Alternative to Antibiotics in Broiler Chickens: Impacts on Growth, Digestibility, and Blood Lipid Profile

**DOI:** 10.3390/vetsci12090860

**Published:** 2025-09-04

**Authors:** Rifat Ullah Jan, Muhammad Ayaz, Shah Zeb Ahmad, Muhammad Tahir, Muhammad Irfan Khan, Muhammad Iftikhar, Huanyong Han, Hosameldeen Mohamed Husien, Zang Yu, Mengzhi Wang

**Affiliations:** 1 College of Animal Sciences and Technology, Yangzhou University, Yangzhou 225009, China; 2 Livestock and Dairy Development Department (Research Wing), Peshawar 25100, Pakistan; 3 Department of Animal Nutrition, Faculty of Animal Husbandry and Veterinary Science, University of Agriculture, Peshawar 25130, Pakistan; 4 State Key Laboratory of Sheep Genetic Improvement and Healthy Production, Xinjiang Academy of Agricultural Reclamation Sciences, Shihezi 832000, China; 5 Laboratory of Metabolic Manipulation of Herbivorous Animal Nutrition, College of Animal Science and Technology, Yangzhou University, Yangzhou 225009, China; 6 College of Veterinary Medicine, Albutana University, Rufaa 22217, Sudan

**Keywords:** moringa oleifera, enrofloxacin, antibiotics, lipid metabolism

## Abstract

Excessive use of antibiotic growth promoters (AGPs) in broiler rearing has led to severe issues due to antimicrobial resistance and drug residues in meat. This study was conducted to evaluate aqueous Moringa oleifera leaf extract (MOLE) as a natural alternative to antibiotics in broiler chickens. 150 broiler chicks were randomly distributed into five groups: one control, three MOLE-treated groups (60, 90, and 120 mL/L), and one Enrofloxacin-treated group (an antibiotic). The birds were monitored for a 35-day trial period, split further into a starter phase (0–21 days) and a finisher phase (22–35 days). The results were that at the starter phase of their lives, birds treated with MOLE120 experienced better body weight gain and optimal feed conversion ratio (FCR), which showed improved early growth performance. In the finisher phase, the MOLE90 group demonstrated the best FCR and a favorable weight gain, showing better efficiency at later stages. Crude protein digestibility was highest in the MOLE90 group (69.97%), and apparent metabolizable energy also increased in all MOLE-treated groups, especially MOLE120 (2938.9 kcal/kg). Regarding the blood lipid profile, the MOLE90 group had the lowest low-density lipoprotein (LDL) (82.3 mg/dL) and cholesterol (181.7 mg/dL), while MOLE120 achieved the highest high-density lipoprotein (HDL) level (92.6 mg/dL) with significant (*p* < 0.05) effects across all parameters. Triglycerides were slightly higher in MOLE groups but remained within physiological limits. In conclusion, MOLE supplementation, particularly at 90–120 mL/L, improved performance and blood lipid metabolism in a phase-specific manner. MOLE120 was more effective in early growth, while MOLE90 proved optimal in the finishing stage. This study supports the potential of MOLE as a phytogenic substitute for antibiotics in poultry production.

## Simple Summary

The excessive use of antibiotics in poultry production contributes to antibiotic resistance and drug residues in meat, posing risks to public health. We explored whether a natural supplement, aqueous Moringa oleifera leaf extract (MOLE), could improve the growth, feed efficiency, and health of broiler chickens as a substitute for antibiotics. Over a 35-day period, we fed different concentrations of MOLE (60, 90, 120 mL/L) to chickens and compared them to an antibiotic-treated group. Our results showed that the 120 mL/L dose improved growth and feed efficiency during the early phase, while the 90 mL/L dose was most effective later. MOLE also improved nutrient digestibility and supported healthier blood lipid profiles, reducing harmful cholesterol and improving beneficial cholesterol levels. Overall, MOLE produced comparable or better results than antibiotics in many respects. These findings suggest that Moringa leaf extract may be a promising, natural alternative to antibiotics in poultry farming, helping to maintain animal productivity while reducing antibiotic use and its associated risks. Furthermore, these findings are valuable to society because they offer a safer way to raise animals for food without depending on antibiotics. Using natural supplements like Moringa leaf extract can lead to healthier meat for consumers, help prevent the spread of antibiotic-resistant bacteria, and support more sustainable farming practices.

## 1. Introduction

Global demand for affordable, quality protein has driven extensive expansion in the poultry industry, and broiler production is now a significant part of food security planning [1]. Not only is poultry meat affordable, but it is also cost-effective in feed conversion, and therefore is a key source of protein, especially in developing countries where per capita consumption of meat is constantly on the increase [2]. According to estimates by Ullah [3], the global population is projected to reach over 9.7 billion by the year 2050, and therefore, it is inevitable that demands will rise for sustainable, scalable, and safe food systems. This demand places significant pressure on broiler farm systems to maximize the efficiency of growth while at the same time meeting health and safety demands.

Antibiotic growth promoters (AGPs) have been used in poultry feed for decades to induce feed efficiency, inhibit pathogenic bacteria, and enhance quick weight gain [4]. The advantages of AGPs have put them at the center of intensive poultry production systems. Nevertheless, the prolonged and sub-therapeutic intake of antibiotics has triggered strong public health concerns due to the emergence of antimicrobial-resistant bacterial strains and the buildup of antibiotic residues in poultry products [5]. Consequently, several countries have placed restrictions or complete bans on the usage of AGPs in food animals. Sweden was the first country to implement a nationwide ban in 1986, and the European Union followed with a blanket ban in 2006 [6]. Pakistan only recently began developing regulatory measures against antimicrobial usage in food animals. The National Action Plan (NAP) against AMR (2017–2022) had a strategic goal of phasing out AGPs and encouraging suitable alternatives. Enhancement is still in its early stages, however, with the majority of interventions remaining in pilot or planning stages, and no official AGP prohibition having yet been enacted in the poultry or livestock industries [7]. The policy reforms, as much as they were needed for public health, have brought new challenges to poultry farmers, most significantly in the form of compromised growth rates, reduced nutrient digestibility, and susceptibility to infection [8].

In Pakistan, the poultry sector is a significant industry in Pakistan’s economy, with a contribution of 12.5% to the livestock sector and 1.4% to the national GDP. The poultry investment was valued at PKR 700 billion during 2017–2018, which indicates its contribution to food security [9].

To combat these challenges, scientists have resorted to nature and safe alternatives to AGPs. Among the promising categories are phytogenic feed additives, which are plant components such as herbs, spices, essential oils, and extracts with antimicrobial, antioxidative, and digestive stimulant activity. Among these, Moringa oleifera is important because it contains rich nutritional and pharmacological compounds, including polyphenols, such as quercetin, catechin, kaempferol, and rutin, which have been shown to enhance the growth, immunity, and intestinal health of poultry [10]. Moringa oleifera boasts a superior nutritional and pharmacological profile. Also referred to as the “miracle tree,” Moringa oleifera naturally grows in South Asia and Africa and is cultivated extensively for human and animal use [11]. The leaves contain flavonoids, alkaloids, phenolic acids, vitamins, and essential amino acids, which confer antimicrobial, anti-inflammatory, and lipid-lowering activity.

Moringa oleifera leaf is nutritionally dense, having around 27–30% crude protein, 6.5–9% fat, and 38–41% carbohydrates on a dry weight basis [12]. It also provides key minerals such as iron (103 mg/100 g), calcium (300 mg/100 g), and zinc (3 mg/100 g), making it a potent poultry feed supplement. Fat content consists of health-benefiting unsaturated fatty acids and oleic, linoleic, and linolenic acids, which may be responsible for enhanced lipid metabolism and cardiovascular health [13]. These bioactive lipids and a broad range of polyphenols, flavonoids, and saponins have been linked to antioxidant, antimicrobial, and gut-modulatory activities. Various studies indicate that Moringa leaves can modulate gut microbiota of poultry by inhibiting pathogens like E. coli and enhancing beneficial flora like *Lactobacillus* spp., finally promoting nutrient utilization and immune status [14].

Several research studies have been done on Moringa oleifera leaf powder growth performances as a feed additive in poultry science. The results show improved growth performance, immune status, and blood lipid metabolism in broilers with no negative impact on feed intake or meat quality. For instance, Djunaidi et al. [15] showed improved weight gain and feed efficiency in broilers with dried Moringa oleifera leaf meal, while Balarabe et al. [16] observed improved carcass quality, including higher dressing percentage, breast, thigh and leg muscle yield, based on traits like dressing weight and prime cuts, along with enhanced blood lipid profiles in broilers given Moringa olifera leaf prime cuts and Moringa olifera leaf powder. Additionally, El-Damrawy [17] showed that supplementation with Moringa improved final body weight, as well as feed conversion ratio (FCR), compared to control. Such observations suggest that Moringa oleifera leaf powder is of high potential as an effective growth promoter in antibiotic-free chicken systems.

Although these findings are encouraging for poultry application, the majority of the investigations were carried out with Moringa dry leaf meal fractions. Other recent research, however, shows that aqueous extracts can produce better performance by increasing water-soluble bioactive bioavailability, as well as easier absorption when delivered in a supplement through drinking water [18]. 

Interestingly, Moringa oleifera aqueous extract and ground leaf were previously shown to be non-toxic to chickens, humans and laboratory animals, proving its safety for incorporation into feeds [19]. This study aims to address these knowledge deficits by comparing the effectiveness of aqueous Moringa oleifera leaf extract (MOLE) as a phytogenic alternative for antibiotics in use in broiler chickens. To this end, this study will evaluate graded levels of MOLE (60, 90 and 120 mL/L) added to broiler drinking water, and will be compared against a positive control that receives the typical antibiotic (Enrofloxacin) and a negative control that receives nothing. Indicators of key performance that will be evaluated include average daily weight gain, feed conversion ratio (FCR), crude protein digestibility, apparent metabolizable energy (AME), and blood lipid parameters, including total cholesterol (TC), triglycerides (TG), high-density lipoprotein (HDL), and low-density lipoprotein (LDL).

The research context of this study reflects the growing realization of a global systemic shift towards consumer-safe and durable broiler systems. Market evidence suggests growing consumer demand for meat without antibiotics, as well as increasing pressure from regulatory bodies to eliminate chemical growth promoters. Meanwhile, Moringa oleifera can offer two benefits, firstly as an immunity and growth promoter, and secondly as a natural meat quality contributor via its blood lipid-modulating effects. In addition to the above, bibliometric reviews by Phillips et al. [20] and Aminullah et al. [21] also demonstrate a meteoric rise in global research interest in the use of phytogenic supplements in broilers, indicating their growing importance to broiler rearing in the commercial sector.

Despite this, the sector is still beset by a number of essential knowledge gaps in the application of phytogenic in animal production, such as uncontrolled dosing regimens, inconsistency in extract forms, and insufficient knowledge about controlling biochemical processes [22,23]. Such gaps are filled in this study by adopting a well-controlled experimental study design that also provides a comparison with MOLE to an established antibiotic regimen. By presenting quantitative information on growth, nutrient uptake, and lipid metabolism indices, this study provides the groundwork for the integration of MOLE into subsequent poultry health and performance programs.

Lastly, the purpose of this study is to demonstrate the efficacy and safety of aqueous Moringa oleifera leaf extract as a substitute for synthetic antibiotics in broiler feeds. It also aims to provide poultry producers with evidence-based recommendations for incorporating natural, plant-based growth promoters into sustainable production systems, thereby aligning animal health strategies with public health and environmental priorities.

## 2. Materials and Methods

### 2.1. Ethical Approval

All Animal experiments were performed in accordance with the guidelines approved by the Animal Care and Use Committee (IACUC) of Yangzhou University, China; Approval Code:202010018, Approval Date: 26 February 2020.

### 2.2. Experimental Design and Birds

A total of 150 day-old mixed-sex broiler chicks (Ross 308) were obtained from a Jadeed hatchery. Upon arrival, birds were weighed and randomly divided into five treatment groups (n = 30 per group), each subdivided into three replicates of 10 birds per pen. Birds were fed with commercial broiler starter (Feed No. 14) and finisher (Feed No. 13) diets obtained from Jadeed Feeds Pvt. Ltd. Khanewal, Punjab, Pakistan. Feed composition and nutrient values were consistent with standard NRC (1994) guidelines. The study spanned 35 days and included a 7-day adaptation period, followed by a 21-day starter phase (Day 0–21) and a 14-day finisher phase (Day 22–35).

The groups were organized as follows:

- MOLE0: Basal diet + 0 mL/L Moringa oleifera leaf extract (Control), which served as the negative control group;
- MOLE60: Basal diet + 60 mL/L MOLE in drinking water;
- MOLE90: Basal diet + 90 mL/L MOLE in drinking water;
- MOLE120: Basal diet + 120 mL/L MOLE in drinking water;
- MOLE0 + AB: Basal diet + antibiotic (Enrofloxacin 10%) in water at 1 mL/L for the first five days (positive control).

Birds were randomly allocated to treatment groups using a random number generator. No blinding was used, and power analysis was not performed due to resource limitations. All birds were housed in a well-ventilated open-sided poultry shed under standard management conditions. Feed and clean drinking water were offered ad libitum, and vaccinations were administered as per local veterinary guidelines. Mortality, if any, was recorded.

### 2.3. Chemical Composition of the Diet

Basal diets employed during the starter (Feed 14) and finisher (Feed 13) stages were commercial broiler diets made as per NRC (1994) recommendations and supplied by Jadeed Feeds Pvt. Ltd. Khanewal, Punjab, Pakistan. Representative proximate composition, according to the manufacturer’s specification, is given in Table 1 below.

### 2.4. Vaccination and Biosecurity

All chicks were vaccinated according to a pre-determined schedule to ensure uniform health management across all groups. The vaccination plan is shown in the below given Table 2.

All vaccines were administered through drinking water, following the manufacturer’s guidelines for dosage and handling. Strict biosecurity measures were maintained throughout the study to prevent disease transmission. These included sterilization of feeding and drinking equipment on a daily basis, maintenance of footbaths at all pen entrances, and restricted access to animal housing areas. Personnel were required to wear protective clothing and follow hygiene protocols before entering the experimental facility.

### 2.5. Preparation of Moringa Oleifera Leaf Extract (MOLE)

Fresh Moringa oleifera leaves were collected in the early spring season from several mature trees from the University of Agriculture, Peshawar, and surrounding areas. Only the mature green, healthy leaves were picked; no fruits, flowers, or young shoot tips were taken. Leaf harvesting was performed in the early morning (7:00–9:00 am) to maximize moisture retention and stability of metabolites. Leaves were shade-dried for 3–5 days at room temperature (28 ± 2 °C) and ground using a mechanical grinder (RETSCH GmbH, Haan, Germany) into fine powder. For extract preparation, 60 g of powdered leaves were soaked in 1000 mL of distilled water for 24 h at ambient temperature. The mixture was filtered through muslin cloth to remove debris, and the filtrate was stored in clean containers for use. MOLE was freshly prepared as needed and diluted to desired concentrations in drinking water (60, 90, and 120 mL/L). The dosage levels (60, 90, and 120 mL/L) were selected based on previous studies [11]. Although in the current study, chemical analysis (e.g., Weende or phytochemical screening) was not conducted, in the future, active metabolites (e.g., flavonoids, tannins, saponins) should be quantified to correlate the biological effects with certain phytochemical components.

### 2.6. Growth Performance

Body weight (BW) and feed intake (FI) were recorded weekly. Birds were not fasted before weighing. Feed Conversion Ratio (FCR) was calculated as

$$\mathrm{FCR}=\frac{Feed\;Intake\;(g)}{Body\;Weight\;Gain\;(g)}$$

Feed intake was determined by subtracting refused feed from the feed offered. Mortality rates were recorded as

$$\mathrm{Mortality}\;\mathrm{Rate}\;(\%)=\frac{Number\;of\;birds\;died}{Initial\;number\;of\;birds}\;\times \;100$$

### 2.7. Nutrient Digestibility

New fecal samples were taken directly from clean plastic-lined trays placed under each pen to avoid contamination. Daily, the samples of all replicates were mixed, labeled, and transported immediately to the laboratory. For dry matter (DM) analysis (Memmert UNB 400), approximately 0.5 g of each sample was pipetted into a pre-weighted crucible and left overnight in a hot air oven at 103 °C. The crucibles were desiccated and reweighed to calculate the percentage of dry matter. Crude Protein (CP) digestibility was determined using the Kjeldahl method, which involved digestion, dilution, distillation, and titration with standardized H_2_SO_4_. The nitrogen percentage (N%) was calculated, and CP was derived using the conversion factor:

Crude Protein (%) = *N*% × 6.25

CP digestibility (%) was calculated as follows:

$$\mathrm{CP}\;\mathrm{Digestibility}\;(\%)\;=\;\frac{CP\;intake-CP\;excreted}{CP\;intake}\;\times \;100$$

Digestible Energy (DE) = GE intake − GE feces

Apparent Metabolizable Energy (AME) was measured using a bomb calorimeter (Parr Instrument Company, Moline, IL, USA, 6400 Calorimeter). Approximately 0.5 g of each dried sample was combusted in a controlled oxygen chamber, and the resulting rise in water temperature was used to compute gross energy value in cal/kg. AME (kcal/kg) was calculated as the difference between gross energy intake and gross energy excreted.

### 2.8. Blood Sampling and Biochemical Analysis

At the end of the experiment (Day 35), 3 mL of blood was collected from the wing vein of each selected bird into tubes containing anticoagulant. Blood samples were centrifuged at 2500 rpm for 10 min to separate the serum, which was stored at −20 °C for further analysis.

Serum biochemical parameters were measured using a semi-automated chemistry analyzer (Microlab-300, Vital Scientific NV (now part of ELITech Group), Dieren, The Netherlands) and commercially available diagnostic kits. The following blood lipid profile parameters were evaluated:

Total Cholesterol (TC) via enzymatic colorimetric method (Cholesterol Oxidase–Peroxidase);

Triglycerides (TG) via Glycerol-3-Phosphate Oxidase method;

High-Density Lipoprotein (HDL) via phosphotungstate precipitation;

Low-Density Lipoprotein (LDL) was calculated using the Friedewald formula:

$$\mathrm{LDL}\;=\;\frac{TG}{5}\;+\;\mathrm{HDL}\;-\;\mathrm{Cholesterol}$$

### 2.9. Statistical Analysis

Data were analyzed using one-way Analysis of Variance (ANOVA) under Completely Randomized Design (CRD) using SAS software (1998 version). Mean comparisons were conducted at a significance level of *p* < 0.05.

## 3. Results

### 3.1. Growth Performance

The growth performance of broiler chickens was significantly influenced by the dietary supplementation of aqueous Moringa oleifera leaf extract (MOLE) at varying concentrations. As shown in Table 3, the highest final body weight was recorded in the MOLE120 group (2149.1 g), representing an 8.3% increase compared to the MOLE0 control group (1984.7 g). This was followed closely by the MOLE0 + AB group (2140.1 g), which showed a 7.8% increase, and the MOLE90 group (2070.2 g) with a 4.3% increase relative to the control. The lowest final weight was observed in the MOLE0 control group (1984.7 g), indicating that the inclusion of MOLE in drinking water improved body weight gain compared to untreated chicks. These relative improvements highlight the potential economic efficiency of MOLE supplementation in broiler production.

Although the analysis of variance did not indicate a statistically significant difference in feed intake across groups (*p* = 0.1503), a clear numerical trend was observed. The control group (MOLE0) consumed the highest amount of feed (3379.7 g), while the MOLE90 group consumed the least (3154.7 g), suggesting more efficient feed utilization in the MOLE-supplemented birds. The MOLE60 and MOLE120 groups had intermediate feed intakes (3263.2 and 3314.0 g, respectively), while the antibiotic group (MOLE0 + AB) recorded a feed intake of 3377.8 g, comparable to the control.

The feed conversion ratio (FCR) results further support this efficiency. The MOLE90 group exhibited the most favorable FCR (1.5237), indicating that the birds required only 1.52 g of feed to gain 1 g of body weight—0.18 points superior to the control (1.7037). This was followed by the MOLE120 group (1.5420), the antibiotic group (1.5780), and MOLE60 (1.6586). The control group had the poorest FCR overall. These findings clearly suggest that 90 mL/L of MOLE provided the most efficient conversion of feed to body weight, comparable to or even superior to the antibiotic-treated group.

No mortality was recorded in any of the experimental groups throughout the trial, reinforcing the safety and tolerance of MOLE even at the highest supplementation level (120 mL/L). These results demonstrate that MOLE, particularly at 90 mL/L, is a promising natural alternative to antibiotic growth promoters, likely enhancing performance through better digestion and nutrient absorption supported by its bioactive compounds.

### 3.2. Weekly Body Weight Gain and Feed Intake Trends

To provide deeper understanding of growth trends during the trial, weekly body weight gain (BWG) and feed intake (FI) data were recorded and are presented in Table 4 and Table 5. A consistent increase in body weight across all treatment groups over the five-week period, with the starter phase defined as Day 0–21 and the finisher phase as Day 22–35. Notably, from the third week onward (Day 15–35), the MOLE90 and MOLE120 groups clearly outperformed the other groups in terms of weekly weight gain.

Notably, from the third week onward, the MOLE90 and MOLE120 groups generally exhibited numerically greater weight gain than the control group. In the final week, both MOLE120 and MOLE90 achieved higher weight gain than MOLE0.

Weekly feed intake remained relatively stable across groups, but MOLE90 tended to consume less feed while achieving greater weight gain, indicating more efficient feed utilization. These weekly trends align with phase-based performance patterns observed in Section 3.1, where MOLE120 was more beneficial in the starter phase, while MOLE90 demonstrated improved efficiency in the finisher phase.

### 3.3. Weekly Water Intake of Broiler Chick (mL/Chick)

The water consumptions were determined weekly, during a five-week period, in five experimental groups: MOLE 0, MOLE 60, MOLE 90, MOLE 120 and MOLE 0 + AB. The values reflect mean consumption of water (units to be stated) per group per week, and the total consumption was computed over the five weeks. A standard error of the mean (SEM) and *p* value of each week and cumulative total are provided. In Week 3, a statistically significant change in water intake between the groups (*p* = 0.0489) was found, with MOLE 0 + AB exhibiting the largest intake of water. No significant differences were observed in other individual weeks and the total water intake (*p* > 0.05). Letters above the bars represent week-by-week group differences, with a different letter assigned to pairs with statistically significant differences.

### 3.4. Nutrient Digestibility

The effect of Moringa oleifera leaf extract (MOLE) supplementation on crude protein (CP) digestibility and apparent metabolizable energy (AME) was evaluated at the end of the experiment (Day 35), and the results are presented in Table 6.

CP digestibility was significantly influenced by MOLE inclusion (*p* = 0.0001). The control group (MOLE0) recorded the lowest digestibility (66.37%), while MOLE120 achieved the highest (70.28%). The MOLE90 group also showed improved digestibility (69.97%), exceeding the antibiotic group (68.21%). These results suggest that MOLE at 90–120 mL/L enhances protein utilization efficiency in broilers.

AME values also showed significant variation among groups (*p* = 0.0028). The control group recorded the lowest energy value (2831.4 kcal/kg), whereas MOLE120 (2957.7 kcal/kg) and MOLE90 (2938.9 kcal/kg) demonstrated higher energy utilization, comparable to the antibiotic group (2960.1 kcal/kg). This indicates that MOLE supplementation at higher concentrations supports improved energy retention.

The improvements in CP digestibility and AME are likely due to the bioactive compounds in MOLE, such as polyphenols and flavonoids, which promote gut health, enzymatic activity, and intestinal absorption.

### 3.5. Blood Lipid Profile

The blood lipid profile of broiler chickens at Day 35 was significantly influenced by the dietary supplementation of aqueous Moringa oleifera leaf extract (MOLE) (Table 7).

Cholesterol levels decreased significantly with increasing MOLE concentrations (*p* < 0.0001), with the lowest values observed in the MOLE120 and MOLE90 groups. Triglyceride (TG) levels showed a slight but significant increase at higher MOLE concentrations (*p* = 0.0001), although all values remained within the physiological range. High-density lipoprotein (HDL) levels were significantly higher in the MOLE120 and MOLE90 groups compared to the control (*p* = 0.0015), supporting the HDL-enhancing potential of MOLE supplementation. Low-density lipoprotein (LDL) levels were significantly reduced in the MOLE120 and MOLE90 groups compared to the control (*p* = 0.0026), indicating a potential hypocholesterolemic effect of MOLE. These findings suggest that higher MOLE concentrations positively influenced serum lipid profiles, with effects comparable to or exceeding those observed in the antibiotic-treated group (Table 8).

## 4. Discussion

This study validated the efficacy of Moringa oleifera leaf extract (MOLE) as a phytobiotic antibiotic alternative in broiler chickens. The findings showed that supplementation with 90 and 120 mL/L MOLE exerted considerable impacts on growth performance, digestibility of nutrients by the gut, and blood lipid profiles, all better than the control and antibiotic-fed treatments. These effects were phase-dependent: 120 mL/L MOLE was more effective during the starter phase, while 90 mL/L showed better efficiency in the finisher phase. Such enhancement suggests that MOLE not only has growth-promoting but also functional metabolic qualities.

### 4.1. Growth Performance and Phytogenic Effects

The results of the present study suggest that Moringa oleifera leaf extract (MOLE) supplementation contributes significantly to the growth performance of broiler chicks. The broilers supplemented with 120 mL/L of MOLE recorded the maximum body weight gain in the starter phase, followed by 90 mL/L, which recorded improved feed conversion in the finisher phase. These findings indicate that optimum performance varied by developmental stage, and MOLE addition to 120 mL/L increased performance metrics with a positive dose–response effect.

Results confirm the earlier work of Abiodun, B.S. et al. [24], who cite that supplementation with up to 120 mL aqueous MOLE remarkably boosted body weight gain following a long duration in broilers. This is attributed to bioactive phytochemicals present in MOLE, like alkaloids, flavonoids, tannins, and saponins, which improve feed efficiency, digestion, and gut health. Zanu et al. [25] further noted increased weight gain as Moringa was incorporated into poultry feed in concentrations of up to 10%.

Interestingly, however, not only did the 120 mL/L MOLE group gain more in initial growth, but they also had a better FCR than all the other groups. These observations indicate that MOLE at 120 mL/L may be better than chemical antibiotics in promoting broiler growth during the initial stage. The improvement at this dose may be due to the synergistic action of MOLE’s bioactive compounds, enhancing digestive secretions and enzymatic activity for better nutrient absorption.

Unlike some research (e.g., Portugaliza et al. and Ashong & Brown [26,27]) which showed improved performance for control groups, the current study noted improved performance in MOLE-treated birds, perhaps because aqueous extract was used instead of dry meal. The results support the efficacy of MOLE as a phytogenic growth promoter, with dose-dependent benefits that manifested differently across the starter and finisher phases.

### 4.2. Mechanistic Insights into Digestibility Enhancement

Digestibility values in the present research indicated that supplementation with Moringa oleifera leaf extract (MOLE) had a remarkable impact on nutrient utilization by broiler chicks, especially dry matter and crude protein digestibility. The 90 mL/L MOLE group had improved a lot, with the 120 mL/L group having superior feed utilization efficiency, as indicated by their excellent feed conversion ratio (FCR).

This rise concurs with previous work by Lannaon [28], who reported that broilers given aqueous Moringa leaf extract had lower feed consumption but improved feed conversion. Here, the greatest feed consumption was in the negative control and antibiotic groups (3379.7 g and 3377.8 g, respectively), and the lowest was in the 60 mL/L MOLE group. Despite lowered feed consumption, birds treated with MOLE at 90 and 120 mL/L showed improved nutrient utilization, confirming the role of Moringa to enhance digestibility as well as metabolic efficiency. This is due to the bioactive constituents within Moringa leaves, such as vitamins, alkaloids, and phenolic compounds such as apigenin, quercetin, and zeatin. All of these work towards mitigating gastrointestinal infections, upholding gut health, and expanding the surface area of absorption by proliferating intestinal villi. The end result is that birds need fewer feeds to achieve their nutritional requirements.

Sidhdhuraja & Becker [29] noted that Moringa has nutrients and antioxidants that ensure nutrient satisfaction and decrease excess feed intake. Likewise, Teixeira, E.M.B. et al. [30] noted that Moringa phenolics ensure gut function, immunity, and metabolic function. This is consistent with our findings that the improved feed conversion at 90 and 120 mL/L MOLE dosages is attributable to improved digestibility and physiological adaptation.

While this study did not measure gut microbiota or biomarkers of oxidative stress directly, the improvements in nutrient digestibility, FCR, and lipid profile that were noted in the MOLE90 and MOLE120 groups could imply an indirect improvement in gut health. Previous studies show that Moringa oleifera contains phytogenic compounds, including flavonoids, saponins, and alkaloids that have antimicrobial and antioxidant activities and can inhibit pathogenic bacteria (e.g., *E. coli*, *Salmonella*) while stimulating beneficial microbes like *Lactobacillus* spp. This possible modification in microbial balance would allow for enhanced nutrient uptake and immunity in broilers. Additionally, enhanced gut integrity and villus morphology—frequently linked to phytogenic supplementation could be the basis for the reported performance advantage. Although microbiological or histological examinations lie outside the scope of this investigation, these results warrant further research into the impact of MOLE on the gut microbial ecology and intestinal structure.

### 4.3. Blood Lipid Profile Modulation and Role of MOLE Bioactives

The findings of this study seemingly reveal that Moringa oleifera leaf extract (MOLE) supplementation significantly affected serum lipid parameters in broiler chicks. Indeed, 120 mL/L MOLE supplementation had the highest values for high-density lipoprotein (HDL) (94.33 mg/dL) and triglycerides (TG) (380.00 mg/dL), and the lowest values for total cholesterol (177.33 mg/dL) and low-density lipoprotein (LDL) (79.33 mg/dL). This implies that MOLE, especially at 120 mL/L, plays a central role in enhancing lipid metabolism and global blood lipid profile.

This is in concurrence with the earlier research works that reported Moringa oleifera’s hypolipidemic activity. As per research, graded dose of MOLE in broilers resulted in lowering total cholesterol and low-density lipoprotein and high-density lipoprotein (HDL) levels. Flavonoids kaempferol and quercetin present in Moringa are known to inhibit cholesterol biosynthesis through HMG-CoA reductase downregulation and enhanced expression of hepatic low-density lipoprotein (LDL) receptors. This effect enables clearance of cholesterol from the circulation, and thus lowers serum lipid levels [31].

Furthermore, the antioxidant activity of Moringa, attributed to vitamin C and polyphenols, is capable of inhibiting blood lipid peroxidation and shielding hepatocytes against oxidative injury. It promotes the metabolism of lipids by ensuring normal function of the liver, which is important for bile secretion, digestion of lipids, and removal of lipoproteins.

These impacts are also validated by the reports of Zanu et al. [32], who obtained similar trends in broilers supplemented with leaf meal of Moringa oleifera. Their reports showed that increased supplementation increased high-density lipoprotein (HDL), decreased low-density lipoprotein (LDL) and total cholesterol, similar to the reports cited in our study.

Thus, MOLE at 120 mL/L has a higher efficacy of controlling blood lipids and has the potential to be utilized as an efficient phytogenic factor for enhancing the blood lipid profile of poultry without any side effects.

### 4.4. Microbiome Interaction Hypothesis

Even though the current research did not explicitly examine the gut microbiota, the performance and health markers indicate that Moringa oleifera leaf extract (MOLE) can have a positive effect on the microbial landscape of the gastrointestinal tract. The improved nutrient digestibility, better feed conversion ratio (FCR), and enhanced blood lipid profile observed, especially in the 90 and 120 mL/L groups, indirectly imply a modulatory effect on intestinal microbial composition.

Previous studies support the hypothesis that phytogenic compounds in Moringa, such as flavonoids, alkaloids, and saponins, exert antimicrobial effects that suppress pathogenic bacteria like E. coli and Salmonella, while simultaneously fostering the growth of beneficial bacteria such as *Lactobacillus* spp [33]. This dual effect likely contributes to improved gut integrity, better nutrient absorption, and enhanced immune response in broilers.

Furthermore, the antioxidant properties of MOLE may reduce gut oxidative stress, which in turn supports the survival of beneficial microbes and protects the mucosal barrier [34]. The resulting gut environment can improve villus architecture, which enhances surface area for absorption, leading to improved feed utilization and performance.

While this study did not include microbial profiling techniques such as 16S rRNA sequencing, the biological outcomes strongly suggest that MOLE contributes to a healthier gut microbiome. Future studies should incorporate microbiological and histological analyses to confirm and clarify the specific microbial pathways influenced by MOLE supplementation.

### 4.5. Comparative Efficacy Versus Antibiotics

The comparison between MOLE-supplemented groups and the antibiotic-fed group reveals that aqueous Moringa oleifera leaf extract (MOLE), particularly at 120 mL/L, performed equally well or even better than antibiotics in terms of body weight gain, feed conversion ratio (FCR), and blood lipid modulation [35]. According to the data from this study, birds receiving 120 mL/L MOLE achieved the highest final body weight (2149.1 g), surpassing even the group supplemented with antibiotics. However, in the finisher phase, 90 mL/L MOLE proved more efficient in FCR than all other groups, showing its suitability for later-stage growth.

Feed intake patterns also attest to the greater efficacy of MOLE. Although the greatest feed intake was noted in the control and the antibiotic-fed groups (3379.7 g and 3377.8 g, respectively), feed intake among the MOLE groups declined with increases in supplementation levels [36]. This shows that enhanced feed efficiency, rather than greater consumption, was responsible for the superior performance, attesting to MOLE’s growth-promoting efficacy without incurring higher feed cost.

Also, the FCR values of birds given 90 mL/L and 120 mL/L MOLE added were significantly improved compared to the control group, with the 120 mL/L group having the most effective feed-to-weight gain ratio. Such performance is due to bioactive compounds in MOLE that facilitate digestion, immune system function, and metabolic activity, which allows for effective utilization of nutrients.

Analysis of blood lipid profile further supports the relative effectiveness of MOLE. Birds in the 120 mL/L MOLE group possessed significantly lower levels of cholesterol (177.33 mg/dL) and LDL (79.33 mg/dL), but higher levels of HDL (94.33 mg/dL) compared to control and antibiotic groups. These improvements in blood lipid metabolism confirm that MOLE can be considered an efficient natural alternative to synthetic antibiotics [37].

Together, these findings highlight that MOLE, especially at 120 mL/L, is a safe and effective replacement for antibiotics. It not only increases growth and physiological performance but also helps towards the eradication of worldwide challenges of antibiotic resistance and drug residues in poultry produce.

### 4.6. Dose Optimization and Threshold Limits

The present study confirmed that the physiological impact of Moringa oleifera leaf extract (MOLE) on broiler chicks depends on the dose [38]. Gain in body weight improved progressively with augmenting levels of MOLE supplementation, and the highest was noted at 120 mL/L. Broilers at this level exhibited the highest final body weight and optimal feed conversion ratio, which reflects that 120 mL/L MOLE resulted in the greatest improvements in performance traits observed under the conditions of this study. These findings indicate that 120 mL/L MOLE supplementation produced the highest performance values among the tested treatments in this experiment and may offer potential as an alternative to antibiotic use.

The findings for this study are in agreement with earlier findings, which reported that supplementation of Moringa oleifera to the extent of 120 mL resulted in increased poultry bird weight gain. Findings from this study are in agreement with Zanu et al. [32], who demonstrated improved performance with the addition of up to 10% Moringa leaves in diets. This dose-dependent rise is presumably because of the bioavailability of phytochemicals like flavonoids, saponins, and terpenoids that boost digestion and metabolic activity.

Contrary to previous phytogenic dose–response studies that show diminishing effects or plateauing at higher concentrations, the current study did not observe any negative effect at 120 mL/L. In fact, this group outperformed even the antibiotic-fed group in several parameters. This suggests that 120 mL/L may represent a biologically optimal dose for MOLE when administered in aqueous form rather than meal-based form, as previously tested by other researchers.

While 90 mL/L MOLE was associated with better feed efficiency in the finisher phase, 120 mL/L produced the highest overall body weight gain during the trial. It demonstrated phase-specific benefits without adverse effects, establishing its potential as a practical and effective replacement for antibiotics in commercial broiler operations.

### 4.7. Study Limitations and Future Directions

Although the current study provides strong evidence of the positive effects of Moringa oleifera leaf extract (MOLE) on broiler growth, nutrient digestibility, and lipid metabolism, it is not without limitations. The mechanisms underlying the observed physiological changes, such as improved weight gain, feed efficiency, and blood lipid profile, were interpreted based on known phytochemical actions rather than directly examined in this trial.

One key limitation is the absence of microbiome analysis. While it is assumed that MOLE influenced gut microbial balance due to its antimicrobial and prebiotic properties, no microbial profiling (e.g., 16S rRNA sequencing) was conducted to validate this effect [39]. Likewise, although the improved feed conversion ratio and digestibility suggest enhanced intestinal morphology and nutrient absorption, histological studies of the gut (such as villus height and crypt depth) were not performed.

Moreover, the study focused solely on physiological parameters and did not include an economic analysis. For MOLE to be commercially adopted as an antibiotic alternative in broiler production, economic modeling is necessary to assess its cost-effectiveness in large-scale operations. Parameters such as return on investment (ROI), feed cost per kilogram of gain, and consumer market perception need further exploration.

Future studies should also explore long-term feeding trials and meat quality outcomes, including factors like lipid oxidation, carcass yield, shelf-life, and organoleptic properties. These will help establish MOLE’s broader utility not just as a growth promoter but also as a meat quality enhancer.

Finally, studies involving gene expression profiling (e.g., hepatic lipid metabolism genes or antioxidant enzyme expression) would provide clearer insights into MOLE’s mode of action at the molecular level.

## 5. Conclusions

This study tested the effectiveness of aqueous Moringa oleifera leaf extract (MOLE) as a potential substitute for antibiotics in broiler production. The test was done by supplementing MOLE at graded levels (0, 60, 90, and 120 mL/L) to broiler chicks with MOLE0 serving as the negative control and MOLE0 + AB (antibiotic-supplemented) to determine their influence on growth performance, feed consumption, feed conversion ratio (FCR), nutrient digestibility, and blood lipid profile.

Based on these observations, supplementation with MOLE at 120 mL/L produced the most substantial improvements in early growth (starter phase), while 90 mL/L was associated with better feed conversion during the finisher phase. These findings reflect the outcomes under the conditions of this study and suggest potential phase-specific effects of MOLE supplementation.

Feed intake decreased with increasing MOLE concentration. The control groups (MOLE0 and MOLE0 + antibiotics) consumed more feed (3379.7 g and 3377.8 g, respectively), while the MOLE60 group showed the lowest intake. Despite this reduction in feed consumption, birds in the 90 and 120 mL/L MOLE groups had improved FCR values, particularly MOLE90 during the finisher stage, indicating enhanced feed efficiency. This suggests that MOLE improves nutrient utilization and reduces feed requirement per unit of body weight gain, depending on the phase of growth.

Digestibility trials further validated this effect, showing enhanced dry matter and crude protein digestibility in the MOLE90 and MOLE120 groups. These improvements can be attributed to the bioactive compounds in Moringa oleifera—such as flavonoids, alkaloids, and polyphenols—which stimulate enzyme activity, support gut health, and suppress harmful gut microflora.

Blood lipid profile analysis revealed that MOLE supplementation had a significant (*p* < 0.05) impact. The MOLE120 group showed the lowest levels of total cholesterol (177.33 mg/dL) and LDL (79.33 mg/dL), while having the highest HDL (94.33 mg/dL) and triglycerides (380.00 mg/dL). These changes indicate a hypocholesterolemic effect of MOLE, supporting cardiovascular health in broilers and potentially improving meat quality. This supports the recommendation to replace synthetic antibiotics with natural MOLE, especially considering rising concerns over antibiotic residues and resistance.

Based on these observations, it can be concluded that MOLE at 120 mL/L is most effective for improving early growth (starter phase), while 90 mL/L is optimal for efficient feed conversion in the finisher phase. The extract serves as a safe, natural alternative to synthetic antibiotics and aligns with global demands for antibiotic-free poultry production. Further commercial trials, economic assessments, and mechanistic studies are recommended to expand its practical applications and acceptance in the poultry industry.

## Figures and Tables

**Table 1:** Proximate composition of commercial starter (Feed 14) and finisher (Feed 13) broiler diets used in the experiment.

Nutrient	Feed 14, Starter Diet (22–35 Days)	Feed 13, Finisher Diet (0–21 Days)
Crude Protein (CP)	19.0–20.0%	21.5–22.5%
Crude Fat	5.0–6.0%	4.0–5.0%
Crude Fiber	3.0–3.5%	3.0–4.0%
Calcium (Ca)	0.9–1.1%	1.0–1.2%
Available Phosphorus	0.4–0.45%	0.45–0.5%
Methionine	0.45–0.5%	0.5–0.55%
Lysine	1.0–1.1%	1.1–1.2%
Sodium	0.17–0.19%	0.18–0.2%
Salt (NaCl)	0.3–0.4%	0.3–0.4%
Vitamin	Trace	Trace
Moisture	<12%	<12%
Metabolizable Energy	3100–3200 kcal/kg	2900–3000 kcal/kg

**Table 2:** Vaccination schedule of experimental broiler chicks, including type of vaccine, disease targeted, route of administration, and remarks.

Age (Day)	Type of Vaccine	Disease Protected	Route of Administration	Remarks
1	Marek’s Disease	Marek’s	SC	Done at hatchery
1	ND, IB	Infectious Bronchitis, Newcastle	Spray or eye drop	Done at hatchery
7	IBD	Gumboro (Infectious Bursal)	Drinking water	Done at farm
10	IB + ND	Infectious Bronchitis, Newcastle	Drinking water	Done at farm
14	IBD	Gumboro (Infectious Bursal)	Drinking water	Done at farm
21	ND	Newcastle	Drinking water	Done at farm
28	FP	Fowl pox	Drinking water	Done at farm

**Table 3:** Growth performance of broiler chickens (Day 7–35).

Group	Final BW (g)	Feed Intake (g)	FCR
MOLE 0	1984.7 ^c^	3379.7 ^a^	1.7037 ^a^
MOLE 60	1966.9 ^c^	3263.2 ^ab^	1.6586 ^a^
MOLE 90	2070.2 ^b^	3154.7 ^b^	1.5237 ^b^
MOLE 120	2149.1 ^a^	3314.0 ^ab^	1.5420 ^b^
MOLE 0 + AB	2140.1 ^a^	3377.8 ^a^	1.5780 ^b^
SEM	37.92912	41.85976	0.034533
*p* value	0.0002	0.1503	0.0022

SEM = Standard Error of Mean; FCR = Feed Conversion Ratio. Values in the same column with different superscripts (a, b, c) are significantly different (*p* < 0.05).

**Table 4:** Weekly body weight gain (BWG) trends (g).

Group	Week 1	Week 2	Week 3	Week 4	Week 5
MOLE 0	350 ^c^	410 ^c^	430 ^c^	450 ^c^	470 ^b^
MOLE 60	360 ^bc^	415 ^c^	440 ^bc^	455 ^bc^	470 ^b^
MOLE 90	370 ^ab^	430 ^ab^	460 ^ab^	480 ^ab^	500 ^a^
MOLE 120	375 ^a^	435 ^a^	470 ^a^	495 ^a^	510 ^a^
MOLE 0 + AB	365 ^ab^	425 ^ab^	455 ^ab^	475 ^ab^	495 ^a^
SEM	4.301163	4.636809	7.141428	8.276573	8.124038
*p* value	0.0037	0.0016	0.0391	0.0021	0.0215

SEM = Standard Error of Mean. Values in the same column with different superscripts (a, b, c) are significantly different (*p* < 0.05).

**Table 5:** Weekly feed intake (FI) trends (g).

Group	Week 1	Week 2	Week 3	Week 4	Week 5
MOLE 0	620 ^a^	660 ^a^	680 ^a^	700 ^a^	720 ^a^
MOLE 60	610 ^b^	645 ^ab^	665 ^ab^	685 ^ab^	715 ^a^
MOLE 90	600 ^bc^	630 ^bc^	650 ^bc^	670 ^bc^	705 ^b^
MOLE 120	615 ^ab^	640 ^ab^	670 ^ab^	690 ^a^	710 ^a^
MOLE 0 + AB	620 ^a^	650 ^ab^	675 ^a^	695 ^a^	720 ^a^
SEM	3.741657	5.00	5.147815	5.147815	2.915476
*p* value	0.0155	0.0012	0.0231	0.0027	0.0137

SEM = Standard Error of Mean. Values in the same column with different superscripts (a, b, c) are significantly different (*p* < 0.05).

**Table 6:** Weekly water intake of broiler chick (mL/chick).

Group	Week 1	Week 2	Week 3	Week 4	Week 5	Total
MOLE 0	891.93 ^ab^	1357.0 ^ab^	2248.9 ^ab^	1748.5 ^a^	2385.4 ^a^	6382.9 ^a^
MOLE 60	907.02 ^a^	1336.9 ^b^	2243.9 ^ab^	1744.7 ^a^	2274.1 ^ab^	6262.7 ^ab^
MOLE 90	821.37 ^b^	1271.5 ^b^	2092.8 ^b^	1742.8 ^a^	2240.6 ^b^	6076.3 ^b^
MOLE 120	859.56 ^ab^	1372.7 ^ab^	2232.3 ^ab^	1763.9 ^a^	2347.3 ^ab^	6343.6 ^ab^
MOLE 0 + AB	876.35 ^ab^	1452.8 ^a^	2329.2 ^a^	1743.5 ^a^	2344.9 ^ab^	6417.6 ^a^
SEM	15.7	22.4	28.6	19.8	34.2	61.7
*p* value	0.13	0.0743	0.0489	0.8725	0.1987	0.1412

SEM = Standard Error of Mean. Values in the same column with different superscripts (a, b) are significantly different (*p* < 0.05).

**Table 7:** Nutrient digestibility across treatment groups.

Group	CP Digestibility (%)	AME (kcal/kg)
MOLE 0	66.37 ^c^	2831.4 ^c^
MOLE 60	66.52 ^c^	2883.4 ^bc^
MOLE 90	69.97 ^b^	2938.9 ^ab^
MOLE 120	70.28 ^a^	2957.7 ^a^
MOLE 0 + AB	68.21 ^b^	2960.1 ^a^
SEM	0.824809	24.91343
*p* value	0.0001	0.0028

SEM = Standard Error of Mean. Values in the same column with different superscripts (a, b, c) are significantly different (*p* < 0.05).

**Table 8:** Blood lipid profile of broiler chickens (Day 35).

Group	Cholesterol (mg/dL)	TG (mg/dL)	HDL (mg/dL)	LDL (mg/dL)
MOLE 0	208.33 ^a^	348.67 ^c^	80.33 ^c^	103.33 ^a^
MOLE 60	199.67 ^b^	356.67 ^c^	87.67 ^b^	88.63 ^bc^
MOLE 90	181.67 ^c^	371.33 ^b^	92.67 ^ab^	82.33 ^c^
MOLE 120	177.33 ^c^	380.00 ^a^	94.33 ^a^	79.33 ^c^
MOLE 0 + AB	192.67 ^b^	369.33 ^b^	92.33 ^ab^	92.59 ^b^
SEM	5.691409	5.566308	2.5379	4.219472
*p* value	0.0000	0.0001	0.0015	0.0026

SEM = Standard Error of Mean. Values in the same column with different superscripts (a, b, c) are significantly different (*p* < 0.05).

## Data Availability

The datasets used and analyzed in the current study are available from the corresponding author on reasonable request.
